# Giant cell granuloma and neurofibroma in the mandible of a patient with neurofibromatosis type 1: a long-term follow-up case report with radiological and surgical aspects and a review of the literature

**DOI:** 10.1186/s12903-024-04543-9

**Published:** 2024-07-14

**Authors:** Oya Barut, Marcel Mukdad, Karin Danielsson, Per Erik Legrell, Mats Sjöström

**Affiliations:** 1 Oral and Maxillofacial Radiology, Umeå University Hospital, Umeå, Sweden; 2 Oral and Maxillofacial Radiology, Department of Odontology, Umeå University, Umeå, Sweden; 3 Oral and Maxillofacial Surgery, Umeå University Hospital, Umeå, Sweden; 4 Orofacial Medicine, Department of Odontology, Umeå University, Umeå, Sweden; 5 Oral and Maxillofacial Surgery, Department of Odontology, Umeå University, Umeå, Sweden

**Keywords:** Giant cell granuloma, Neurofibromatosis type 1, MRI, Long-term follow-up

## Abstract

**Background:**

Magnetic resonance imaging (MRI) of the brain is frequently performed on patients with neurofibromatosis type 1 (NF1), to detect and follow-up intracranial findings. In addition, NF1-related pathologies can appear in the jaws. This case study investigates if it is advantageous to assess the depicted parts of the jaws in the imaging of NF1 patients with intracranial findings, thereby detecting jaw pathologies in their initial stages.

**Case presentation:**

We report on the 3-year management with clinical and radiological follow-ups of a central giant cell granuloma and a neurofibroma in the mandible of a patient with NF1 who underwent examinations with brain MRIs. A review of the mandible in the patient’s MRIs disclosed lesions with clear differences in progression rates.

**Conclusion:**

NF1-related jaw pathologies may be detected in the early stages if the depicted parts of the jaws are included in the assessment of the imaging of NF1 patients with intracranial findings. This could impact the treatment of eventual pathologies before lesion progression and further damage to the vicinity.

## Background

Neurofibromatosis type 1 (NF1) is a genetic disorder that is caused by mutations in the NF1 tumor-suppressor gene [1]. The birth incidence has been estimated as 1 in 2,700 and the disease prevalence in order of frequency has been reported as 1 in 4,560 [2]. Approximately half of the patients show *de novo* mutations in the absence of a family history of the disease [1].

Pigmentation abnormalities (such as café-au-lait macules and freckling) in the axillary or inguinal regions, benign Schwann-cell tumors (neurofibromas), plexiform neurofibromas that have a risk for malignant transformation, iris hamartomas, skeletal abnormalities (such as osteopenia and scoliosis), and nervous system tumors (especially, optic pathway glioma) are the main clinical characteristics of NF1 [1, 3]. There is a well-established diagnostic criterion for diagnosing NF1 that mainly takes into account the clinical features, although in some unusual cases NF1 genetic testing may be needed for a definitive diagnosis [1].

People with NF1 have an increased risk to develop benign and malignant tumors, especially in the head and neck region and in the nervous system [4, 5]. The lifetime risk for glioma of the optic pathway is reported as being 15–20%, and the risk for other brain tumors is more than five-fold higher [1]. Focal abnormal signal intensity, also known as focal areas of signal intensity (FASI), is another NF1-associated finding in magnetic resonance imaging (MRI) of the brains of patients with NF1 [6–9]. The frequency of FASI in children with NF1 has been reported to be more than 90%, and FASI have been suggested as an additional diagnostic criterion for younger children [10]. Examinations and follow-ups of NF1 patients with brain MRI based on NF1-related intracranial findings are common in clinical practice [6, 7, 11].

NF1-related pathologies in the jaws, oral soft tissue changes, and craniofacial abnormalities that can affect function and speech have been reported in the literature [12–14]. One study reported that 28% (29 out of 102 patients) of the patients with NF1 had radiological findings in the jaws; a common finding was widening of the mandibular canal bilaterally or unilaterally, with disappeared or diminished borders of the mandibular canal making identification difficult [12]. Furthermore, changes in the size and form of the ramus, corpus and condyle of the mandible and concomitant malocclusions and fibro-osseous, dysplasia-like changes of the jawbone are examples of other radiological changes seen in these patients [13]. NF1-related neurofibromas in the jaw usually originate from the soft tissue in the oral cavity [14, 15]. Intraosseous neurofibroma in the jawbone is a rare finding in patients with NF1 [15].

Central giant cell granuloma (CGCG) is a benign intraosseous lesion of unknown etiology. CGCG of the jaws has in recent studies and case reports been linked to NF1 and described as neoplastic in nature, following the detection of specific mutations in the *NF1* gene in the giant cell lesions of patients with NF1 [16–18]. Various treatment strategies for CGCG, such as conservative curettage of the lesion, radical surgical therapy, intralesional injection of corticosteroids, and combination therapies have been presented and discussed in the literature both for the NF1- and other syndrome-related lesions and for the sporadic lesions [17–22].

In earlier studies, pathologies such as CGCG, neurofibroma and cemento-osseous dysplasia have been observed in the jaws of persons with NF1 [12, 15, 22]. Here, we describe a case with two different pathologies, neurofibroma and CGCG, in the anterior mandible of a patient with NF1, presenting both the surgical and radiological aspects and providing a review of the relevant literature. The aim of this case presentation is to investigate if it can be beneficial to include assessments of already depicted parts of the jaws in follow-up MRIs of patients with NF1 complications in the brain. For this, we consider the risk of developing NF1-related jaw pathologies and the early detection of these pathologies. Furthermore, we present the treatment strategy for an NF1-related CGCG and describe the 3-year follow-up of the patient.

## Case presentation

A 29-year-old woman was referred to the Orofacial Medicine Clinic from the Dermatology Clinic at the University Hospital of Umeå in December 2020. The patient had NF1 and was being followed up at the dermatology clinic for multiple neurofibromas on the skin and in the breast. The patient had multiple FASI, which were examined with brain MRIs. Furthermore, the patient had undergone surgery for scoliosis.

The reason for the referral was that the patient complained about recurring pain and tenderness in the lower-right second incisor and the lower canine on the same side. She experienced that the incisor was “loose” and felt that “the gum in this region looked bluish”.

### Clinical and imaging findings

**Fig. 1:**
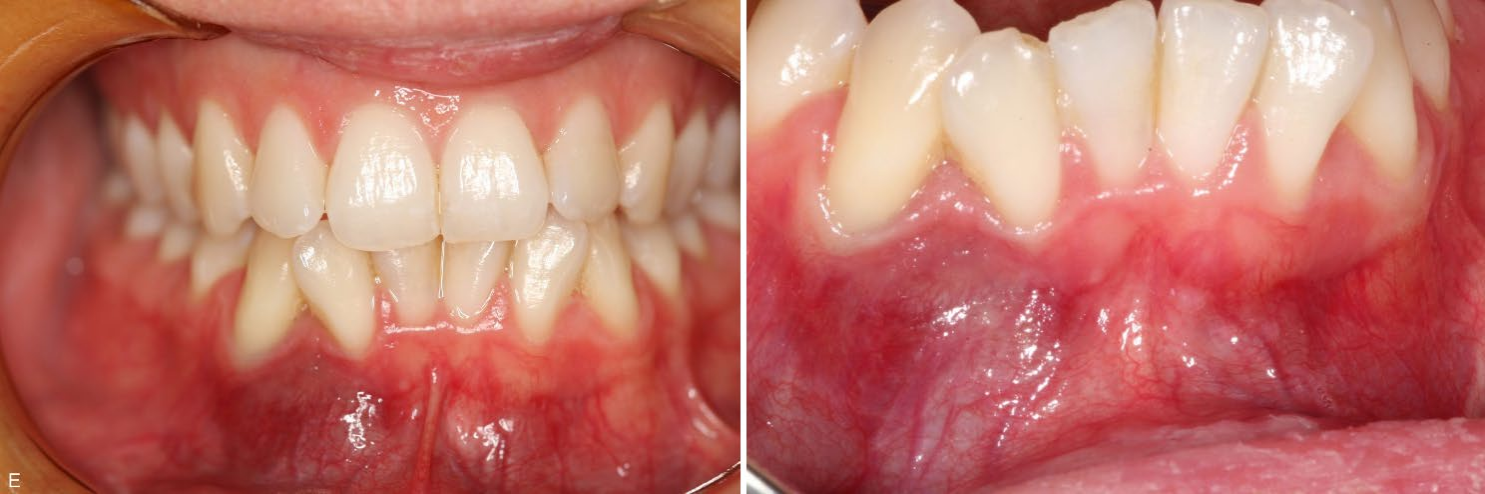
The photographs reveal slight swelling and bluish coloration of the gum buccally in the region of the second incisor and the canine on the right-hand side, as compared with the corresponding area on the left-hand side

The examination of the patient at the Orofacial Medicine Clinic showed slight swelling with a slightly bluish coloration of the gum in the region of the lower-right second incisor and the canine (Fig. 1).

The buccal gingiva was found to be soft on palpation. A deep gingival pocket, about 8 mm in depth, was measured distally to the second right incisor, which was slightly mobile and percussion-sensitive. After the clinical examination, the patient was sent to the Oral and Maxillofacial Radiology Clinic for further examination.

**Fig. 2:**
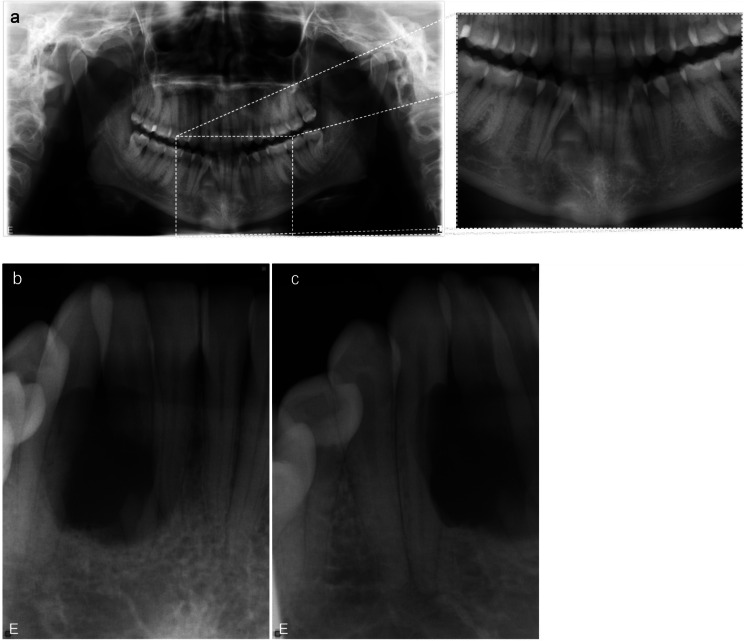
An osteolytic lesion is seen in the interdental bone between the second incisor and the canine, which appear to be extensively resorbed on the panoramic (**a**) and intraoral radiographs (**b**, **c**)

Previous bitewing radiographs were accessible in the medical and/or dental records, and no other radiological examinations of the teeth and jaws were available. The current radiological examination started with a panoramic image, to obtain an overview of the jaws; no jaw deformities could be identified. There were no signs of enlargement of either the mandibular canal or mandibular foramen in the mandible. The patient had all the permanent teeth, including completely erupted wisdom teeth. The panoramic image suggested loss of the trabecular structure of the interdental bone in the region of the abovementioned teeth. Obvious root resorptions and mild deviation of these teeth were observed (Fig. 2a). The radiological examination was completed with intraoral radiographs, to enable comparisons at follow-ups of the patient. The intraoral examination revealed an osteolytic lesion with diameter of approximately 1.5 cm. Extensive root resorptions in the second incisor and the canine were clearly evident (Fig. 2b-c).

**Fig. 3:**
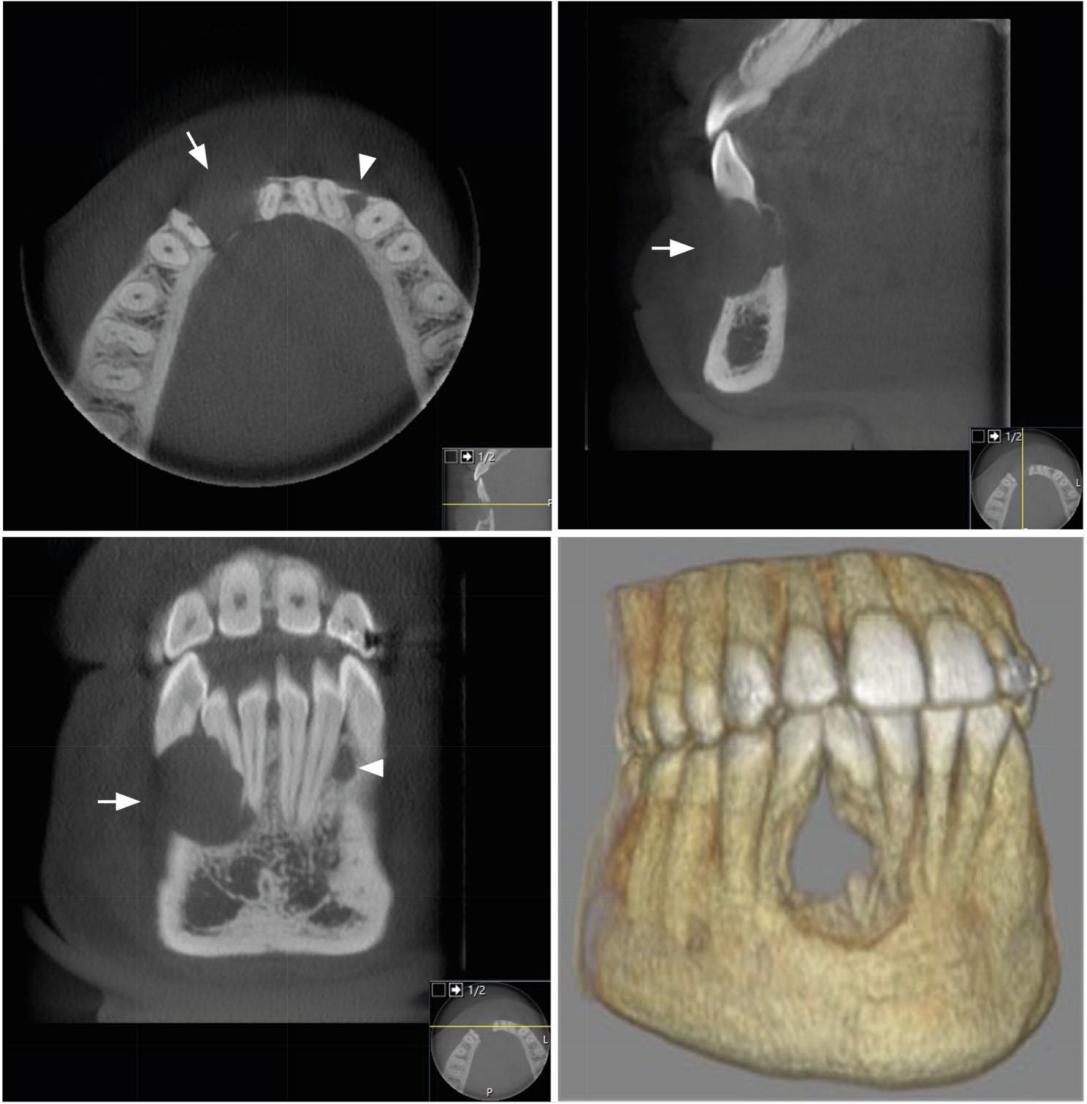
Cone beam computed tomography images showing a well-defined, approximately 1 cm in diameter, radiolucency with destruction of the buccal and lingual cortex in the right anterior mandible and extensive resorptions of the teeth. This lesion is a CGCG (arrow). In the left anterior mandible, another well-defined, approximately 2 mm in diameter, radiolucency with a probable perforation in the buccal cortex is evident. This was a neurofibroma (arrowhead). The 3D reconstruction shows the destruction in the mandible in the area of the CGCG lesion

Cone beam computed tomography (CBCT) of the anterior mandible was performed after scrutiny of the pathologic findings in the panoramic and the intraoral radiographs. The CBCT examination provided detailed information on the pathology on the right-hand side and revealed an additional osteolytic lesion in the interdental bone in the region of the second incisor and the canine on the left-hand side (Fig. 3).

This lesion on the left-hand side was totally separate from that on the right-hand side. Both lesions were suggested to be benign based on their radiological characteristics. The diagnosis of NF1 spurred consideration of a tentative conclusion of the presence of two separate neurofibromas in the anterior mandible.

**Fig. 4:**
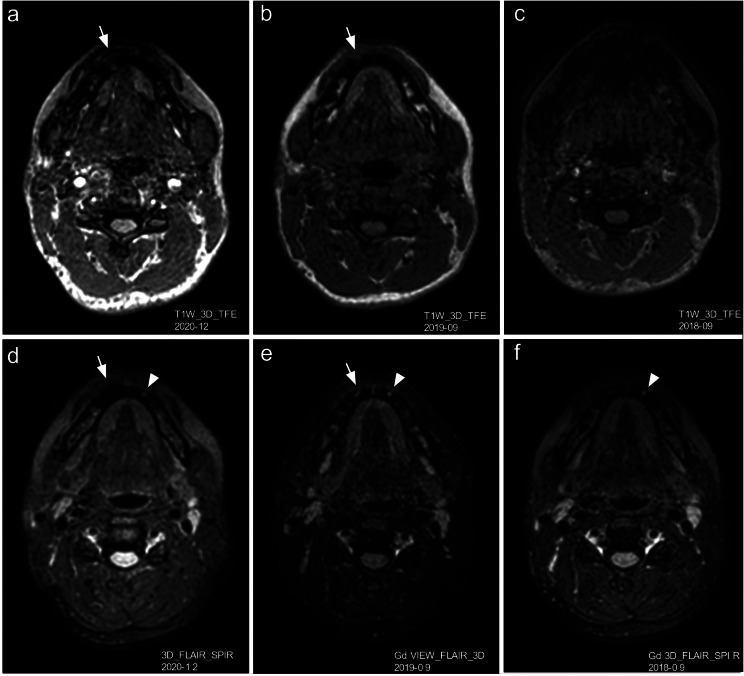
Analyses of axial slices from multiplanar reconstructions. (**a, d**): Rapid volume progression of a finding, with buccal and lingual breakthroughs in the right anterior mandible where (**b, e**); only a buccal discontinuity was seen in the MRI performed 1 year earlier (arrows). (**c**): No pathology is noticeable in the MRI performed in Year 2018. (**d-f**): A high signaling well defined area of the same size over 2 years is seen in the anterior mandible on the left-hand side (arrowheads)

A subsequent review of the mandible in multiplanar reconstructions of the patient’s brain MRIs from September 2018, September 2019, and December 2020 disclosed the development of these lesions over time. The lesion that caused tooth resorptions on the right-hand side had occurred sometime in the previous 2 years and showed rapid expansion of volume in the period from September 2019 to December 2020, while the lesion on the left-hand side was unchanged. Furthermore, the lesions showed different signal patterns in MRIs (Fig. 4).

The conclusion was reached that these two lesions were probably not of the same origin. A differential diagnosis of CGCG was suggested for the lesion on the right-hand side. The patient was referred to the Maxillofacial Surgery Clinic.

### Surgery, diagnosis and treatment

**Fig. 5:**
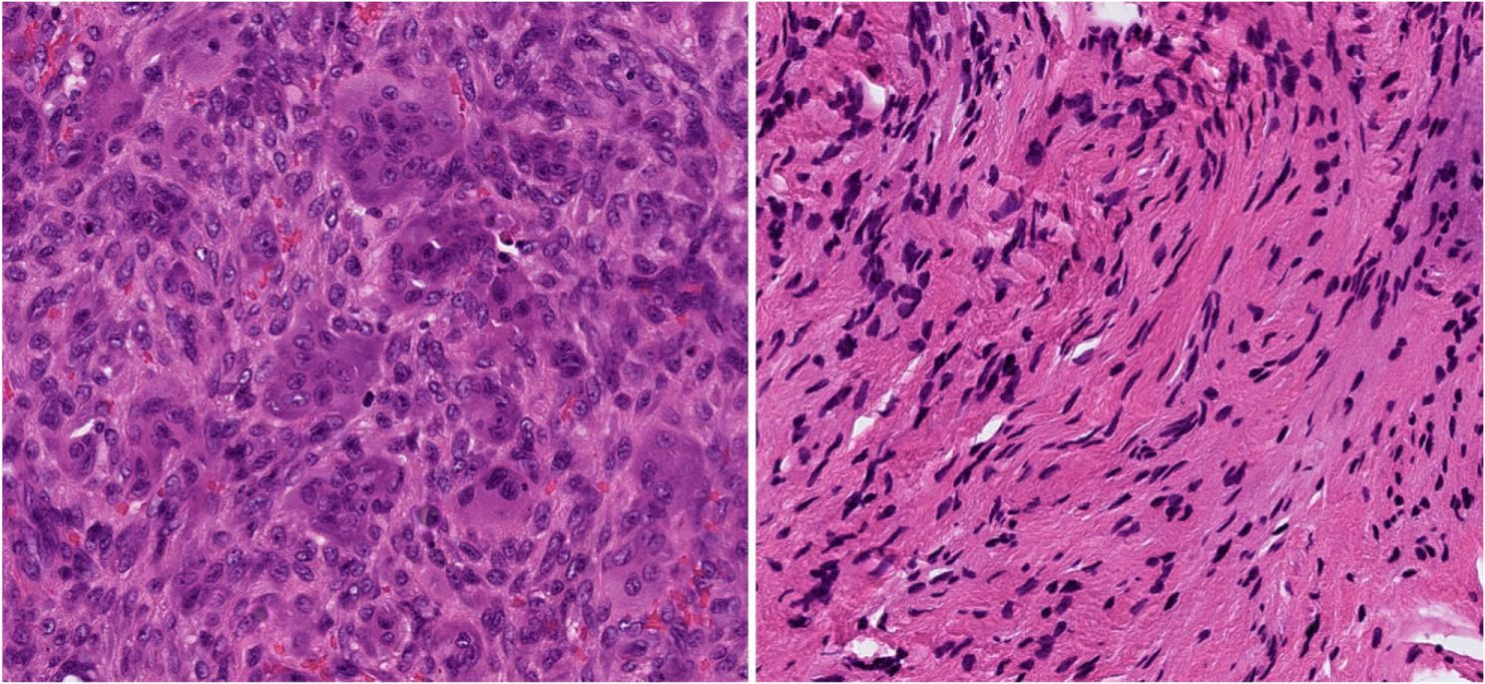
Immunohistochemistry was performed with hematoxicillin eosin staining. Left panel: Photograph of the giant cell granuloma located in the right anterior part of the mandible, showing giant cells and some bleeding in a cell-dense background. Right panel: Photograph of the lesion located in the left anterior mandible, showing a resemblance to the patient´s previously detected neurofibroma

The patient was unwilling to have teeth adjacent to the lesion removed, which resulted in a surgical intervention that was primarily aimed at biopsy. Both lesions were available for easy extirpation as a whole, so a conservative treatment strategy with extirpation and short-interval follow-up was decided upon. Histologic examinations of the lesions showed a CGCG in the right anterior mandible and soft tissue foci with features of neurofibroma in the left anterior mandible (Fig. 5).

The DNA and RNA samples from the CGCG lesion were analyzed for genomic aberrations using a broad next-generation sequencing panel, which included more than 560 cancer-related genes. The analysis determined two inactivating mutations in the *NF1* gene: c.3871–2 A > G and p.Q786fs*30, indicating a loss of function. The result of the analysis was evaluated as not to have a known relevance for the treatment.

**Fig. 6:**
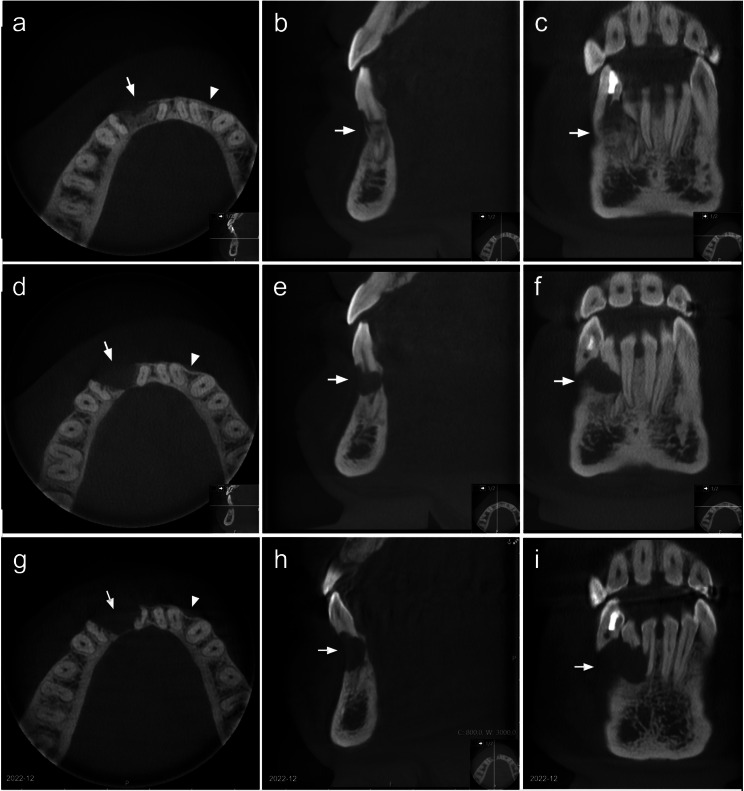
(**a-c**): Cone beam computed tomography image showing bone healing in the areas of both the CGCG (arrow) and neurofibroma (arrowhead) at the 6-month follow-up. (**d-f**) Resorption of the regained bone in the area of the CGCG (arrow), whereas further normalization of the bone is evident in the area of the neurofibroma (arrowhead) at the patient’s 16-month control. (**g-i**) Radiologic follow-up at 6 months following intralesional corticosteroid injection after the first recurrence shows newly established resorption of the first incisor on the right-hand side and an increase in the volume of the osteolytic area (arrow)

Thereafter, the patient was referred to the Department of Endodontics for evaluation of the teeth that had extensive root resorptions in the vicinity. After examinations and discussions regarding the resorbed teeth, endodontic treatment of the canine was carried out, to prevent possible infections of the weakened bone in this area. The incisor was extensively resorbed and mobile grade 2, so no attempt at endodontic treatment was made for this tooth. At the 6-month follow-up with CBCT (Fig. 6a-c) and at the 1-year follow-up, the clinical and radiological examinations were unremarkable, with signs of bone healing after both pathologies. The patient experienced symptoms in the area of the CGCG 4 months after the 1-year follow-up, and the radiographic examination indicated recurrence of the CGCG (Fig. 6d-f).

**Fig. 7:**
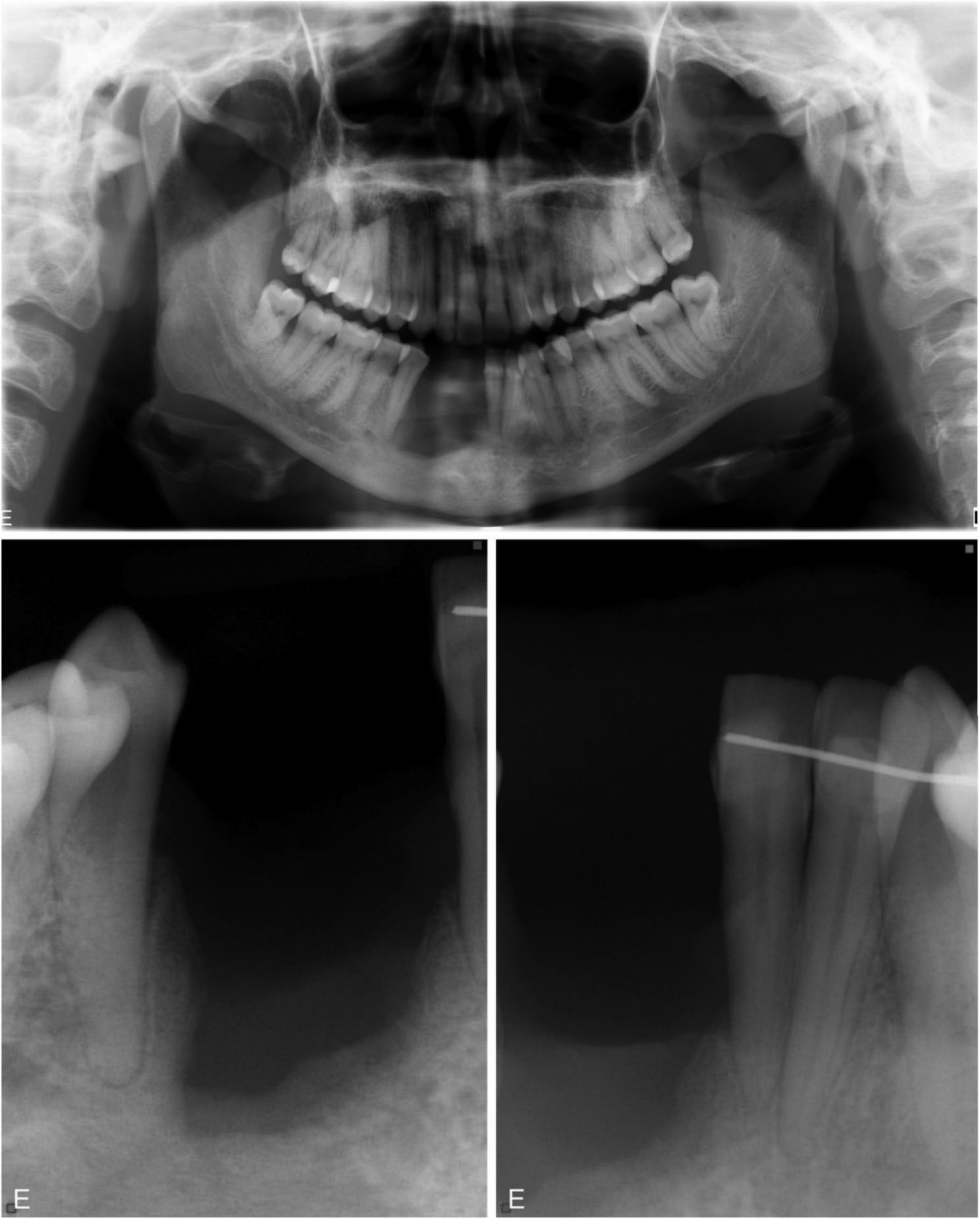
Radiological examination of the patient 6 months after resection was uneventful

The injection of corticosteroid into the lesion was considered as a continuation of the conservative approach, since the patient had no concomitant illnesses that might be contraindicative for corticosteroid administration. The corticosteroid injection was administered after consensus was reached in the multidisciplinary conferences and after discussion with the patient. The therapy was administered as described by Jacoway et al. [23]. The injection solution was prepared by mixing triamcinolone acetonide (Kenacort^®^-T 40 mg/mL; Bristol-Myers Squibb) with levobupivacaine hydrochloride (7.5 mg/mL; AbbVie) in a 1:4 ratio. Over a 6-week period, injections were applied once a week using a 5-mL syringe with a 20-gauge needle. The patient reported no pain or other discomfort during the treatment. To monitor the treatment response, clinical and radiological follow-up was conducted after 6 months. The radiological follow-up indicated a new resorption in the first incisor on the right-hand side and an increase in the volume of the osteolytic area (Fig. 6g-i). Following 2 years with several clinical and radiological follow-ups and the finding of recurrent CGCG, a more-radical treatment with resection of the alveolar process, including the first incisor and the canine on the right-hand side, was performed. The clinical and radiological examinations at 6 months post-resection were unremarkable, showing no signs of recurrence (Fig. 7). Examination at the 1-year follow-up showed stable clinical and radiological statuses. The current plan is to reconstruct the jawbone and to replace the missing teeth.

## Discussion and conclusions

NF1 is a genetic disease in which the affected person has an increased risk of developing benign and malignant tumors, especially in the head and neck region [5]. A recent literature review has reported that FASI are seen in 90% of lifelong NF1 patients [24]. Although the number, size and the clinical relevance of FASI have been reported to decrease with age, and FASI have been regarded as hamartomas and irrelevant with respect to the clinical management of patients, intensive clinical follow-ups and repeated brain MRIs are commonly employed for patients with NF1 [24]. In an earlier radiology-based study, the frequency of jaw lesions in patients with NF1 was reported to be 28% [12]. CGCG is clearly associated with NF1 [16, 17], and it can show rapid growth and local aggressiveness, which may entail radical treatments, such as bone resection, and result in loss of teeth [25]. Furthermore, the need for personalized follow-ups for patients with NF1 and NF1-like disorders has been mentioned in the literature [5]. This case report presents two different pathologies: CGCG and neurofibroma in the mandible of a patient with NF1 who underwent several brain MRIs over time to observe the status of FASI. We hypothesize that patients with NF1 who undergo MRI of the brain would benefit from including the assessment of already depicted parts of the jaws in the MRI examination, so as to facilitate early detection of pathology.

It has been suggested that CGCG can behave aggressively or non-aggressively depending on the clinical characteristics [21, 25, 26]. For aggressive CGCG, the lesions show a rapid increase in volume, recurrence, malocclusion or displacement of the teeth, and root and cortical bone resorptions, as compared with non-aggressive CGCG, which manifests painless and slow growth without tooth or cortical bone resorption [21, 25, 26]. Various treatment methods for CGCG, including conventional surgical methods (curettage, enucleation), radical surgery with resection, and different intralesional medicament injections (e.g., interferon and corticosteroids), have been introduced and discussed for both aggressive and non-aggressive cases [19, 21, 25]. A combination of different treatment modalities has been reported for cases with recurrence of the lesion [27]. A conservative approach with intralesional corticosteroid injections to minimize recurrences and morbidity has been tested as a primary or adjunctive pre-surgical treatment with reported good results, especially for cases of non-aggressive CGCG [28, 29]. Surgical treatment of recurrent NF1-related CGCGs has been reported in the literature as yielding various outcomes, with some cases requiring several rounds of surgical intervention [17, 18]. In the present study, we report on a patient who was diagnosed with recurrent NF1-related CGCG in the mandible and who was treated with a step-by-step treatment approach with frequent clinical and radiological follow-ups. This step-by-step approach started with a conservative surgical excision. Due to the risk of recurrence and with resorbed teeth in the area, frequent clinical and radiological controls were conducted. After the recurrence at 16 months, treatment with intralesional corticosteroid injections was initiated. This type of conservative strategy of treatment, as opposed to an initial radical treatment, can be considered as appropriate, given that patients with NF1 have low bone mineral densities and an increased risk for fractures [1].

A radical approach involving resection of the jawbone was taken following the second recurrence of CGCG. To the best of our knowledge, this is the first report of such a combination therapy for a patient with NF1-related CGCG with 3-year follow-up. Intralesional corticosteroid injection did not produce bone healing in our case. This outcome is discrepant with the results for earlier published cases, for whom it was reported that there was bone healing of the osteolytic CGCG lesions, which were described as non-aggressive and not NF1-related [28, 29]. A positive result following exclusively conservative surgical curettage in an NF1-related CGCG case has been reported previously, with unremarkable findings at the 7-month follow-up [22]. In our study case, following conservative surgical excision, a CGCG recurrence occurred after 16 months. This result is consistent with that of an earlier case report [18].

Central giant cell granuloma has been reported and genetically proven to be associated with NF1 [16, 17]. In recent studies, the genetic analyses have uncovered mutations in the *NF1* gene, suggesting a neoplastic character for CGCG of the jaws in patients with NF1 [17, 18]. In the present case, the genetic analysis revealed two inactivating mutations in the *NF1* gene in the surgically extirpated lesion, which was histopathologically proven to be CGCG.

Neurofibroma in the jawbone has been described as a very rare finding, as compared with oral neurofibromas in the soft tissue of patients with NF1 [15]. In this study case, an intraosseous neurofibroma in a patient with NF1 and with 3 years of follow-up following conservative surgical excision is presented. To our knowledge, there are no published case reports presenting two different pathologies, neurofibroma and CGCG, in the same jaw of a patient with NF1.

Craniofacial changes, oral soft tissue abnormalities, and radiological findings in the jaws of patients with NF1 have been reported [12–14]. Multidisciplinary care of patients with NF1, within which regular dental visits play an important role regarding the risks for abnormalities/changes and developing pathologies, has been reported [13, 14]. The patient in the present study had been visiting a dentist regularly and had only bitewing examinations, which did not show the jaws and the jawbone in general. A multidisciplinary approach wherein the dentists are included in the follow-up strategy for the patients with NF1 could have a significant impact on the treatment of eventual pathologies, preventing the lesion from growing using appropriate radical treatment, and thereby avoiding tooth loss in a young patient, as presented in this case report.

In conclusion, there is a potential benefit associated with including the depicted jaws in the assessment and evaluation of follow-up MRI images of patients with NF1 complications in the brain, in that it can increase the chances for early detection and identification of jaw pathologies. Frequent clinical and radiological follow-ups during, and long after, treatment of NF1-related CGCG in the jaws are recommended. A multidisciplinary approach to patients with NF1 is beneficial considering the risks of developing NF1-related pathologies in the jaws.

## Data Availability

The datasets generated and analyzed during the current study are not publicly available due to Swedish journal act but are available from the corresponding author on reasonable request.

## Abbreviations

NF1: Neurofibromatosis type 1
FASI: Focal abnormal signal intensity, also known as Focal areas of signal intensity
MRI: Magnetic resonance imaging
CGCG: Central giant cell granuloma
CBCT: Cone beam computed tomography
